# An inducible *recA* expression *Bacillus subtilis* genome vector for stable manipulation of large DNA fragments

**DOI:** 10.1186/s12864-015-1425-4

**Published:** 2015-03-18

**Authors:** Takafumi Ogawa, Tetsuo Iwata, Shinya Kaneko, Mitsuhiro Itaya, Junji Hirota

**Affiliations:** Department of Bioengineering, Graduate School of Bioscience and Bioengineering, Tokyo Institute of Technology, Yokohama, 226-8501 Japan; Department of Molecular Bioscience, Graduate School of Bioscience and Bioengineering, Tokyo Institute of Technology, Yokohama, 226-8501 Japan; Institute for Advanced Biosciences, Keio University, Tsuruoka, 997-0017 Japan; Center for Biological Resources and Informatics, Tokyo Institute of Technology, 4259-B63 Nagatsuta-cho, Midori-ku, Yokohama, 226-8501 Japan

**Keywords:** *Bacillus subtilis*, BGM vector, Genome engineering, RecA, Homologous recombination

## Abstract

**Background:**

The *Bacillus subtilis* genome (BGM) vector is a novel cloning system based on the natural competence that enables *B. subtilis* to import extracellular DNA fragments into the cell and incorporate the recombinogenic DNA into the genome vector by homologous recombination. The BGM vector system has several attractive properties, such as a megabase cloning capacity, stable propagation of cloned DNA inserts, and various modification strategies using RecA-mediated homologous recombination. However, the endogenous RecA activity may cause undesirable recombination, as has been observed in yeast artificial chromosome systems. In this study, we developed a novel BGM vector system of an inducible *recA* expression BGM vector (iREX), in which the expression of *recA* can be controlled by xylose in the medium.

**Results:**

We constructed the iREX system by introducing the xylose-inducible *recA* expression cassette followed by the targeted deletion of the endogenous *recA*. Western blot analysis showed that the expression of *recA* was strictly controlled by xylose in the medium. In the absence of xylose, *recA* was not expressed in the iREX, and the RecA-mediated recombination reactions were greatly suppressed. By contrast, the addition of xylose successfully induced RecA expression, which enabled the iREX to exploit the same capacities of transformation and gene modifications observed with the conventional BGM vector. In addition, an evaluation of the stability of the cloned DNA insert demonstrated that the DNA fragments containing homologous sequences were more stably maintained in the iREX by suppressing undesirable homologous recombination.

**Conclusions:**

We developed a novel BGM vector with inducible *recA* expression system, iREX, which enables us to manipulate large DNA fragments more stably than the conventional BGM vector by suppressing undesirable recombination. In addition, we demonstrate that the iREX can be applied to handling the DNA, which has several homologous sequences, such as multiple-reporter expression cassettes. Thus, the iREX expands the utility of the BGM vector as a platform for engineering large DNA fragments.

**Electronic supplementary material:**

The online version of this article (doi:10.1186/s12864-015-1425-4) contains supplementary material, which is available to authorized users.

## Background

As genome analysis progresses, large genome regions including noncoding DNA sequences are drawing much attention. For studies of such regions, technological developments for handling large DNA fragments are essential. Currently, there are several tools available for manipulating large DNA fragments, including bacterial artificial chromosomes (BACs) [1] and yeast artificial chromosomes (YACs) [2]. BACs are based on the F-factor of *Escherichia coli* and can accommodate genomic DNA inserts of up to 300 kb. BAC clones are easy to manipulate and retrieve because of their plasmid form and the stability of the cloned DNA. However, YACs can accommodate larger DNA inserts than BACs. Although the cloning capacity of YACs is extremely large, up to 2 Mb, YAC DNA is difficult to purify because of its linear form, and it suffers from insert chimerism [3,4].

The *Bacillus subtilis* genome (BGM) vector system has been developed as a novel cloning system for handling large DNA fragments [5–7]. *B. subtilis* can import extracellular DNA molecules into the cytoplasm in a single-stranded form through its transformation machinery, and the recombinogenic DNA is then integrated into the genome via RecA-mediated homologous recombination [8]. These sequential events are called “natural competence”. Based on this natural competence, the *B. subtilis* genome can serve as a vector in the BGM vector system. The BGM vector system has several attractive properties, including a large cloning capacity of over 3 Mb, the propagation of cloned DNA fragments in a single copy per cell and the facility of various modification strategies. To date, various types of genomic DNA inserts, including cyanobacteria, *Arabidopsis* and mouse, have been cloned into the BGM vector [5–7,9].

Recently, we have established complete gene modification strategies, including targeted insertion, deletion, inversion and fusion of DNA fragments, and we have applied the BGM vector system to mouse transgenesis [10]. Using the BGM vector system, we reconstructed a 252 kb genomic structure by fusing two mouse genomic DNA fragments of 114 kb and 220 kb in the BGM vector and demonstrated the production of the transgenic mouse carrying the reconstructed DNA. Thus, the BGM vector system can now be recognized as a third platform for transgenesis, in addition to the BAC and YAC systems. Because of the flexibility of the modification strategy and the megabase-scale cloning size, the BGM vector is a promising tool for handling large DNA fragments.

However, the conventional BGM vector system has a potential instability in the cloned DNA inserts. Various gene manipulations in the BGM vector depend on the RecA-mediated homologous recombination. Thus, the endogenous RecA may cause undesirable recombination if there are homologous sequences in the cloned DNA. In fact, undesirable recombination, such as deletion due to the endogenous recombinases, has been reported in the YAC system, which also utilizes the endogenous recombinases for gene modifications [4,11]. One method for preventing such undesirable recombination is to induce the expression of the recombinase specifically during gene manipulations. In the BAC modification strategy that uses the Red system, the recombination proteins are inducible, and the host *E. coli* is *recA*-deficient to stably maintain the BAC DNA [4,12–14]. Accordingly, undesirable recombination can be prevented by the introduction of an inducible system into the BGM vector system.

In this study, we developed an inducible *recA* expression BGM vector (iREX) by introducing a xylose-inducible *recA* expression cassette and deleting the endogenous *recA*. Western blot analysis showed that the expression of *recA* was strictly controlled by xylose in the medium. In addition, we demonstrated that stability of the cloned DNA is improved in the iREX in the absence of xylose by suppressing the *recA* expression. Our novel BGM vector, iREX, offers a new platform for stable gene manipulation of large DNA fragments.

## Results and discussion

### Construction of the inducible *recA* expression BGM vector (iREX)

**Figure 1:**
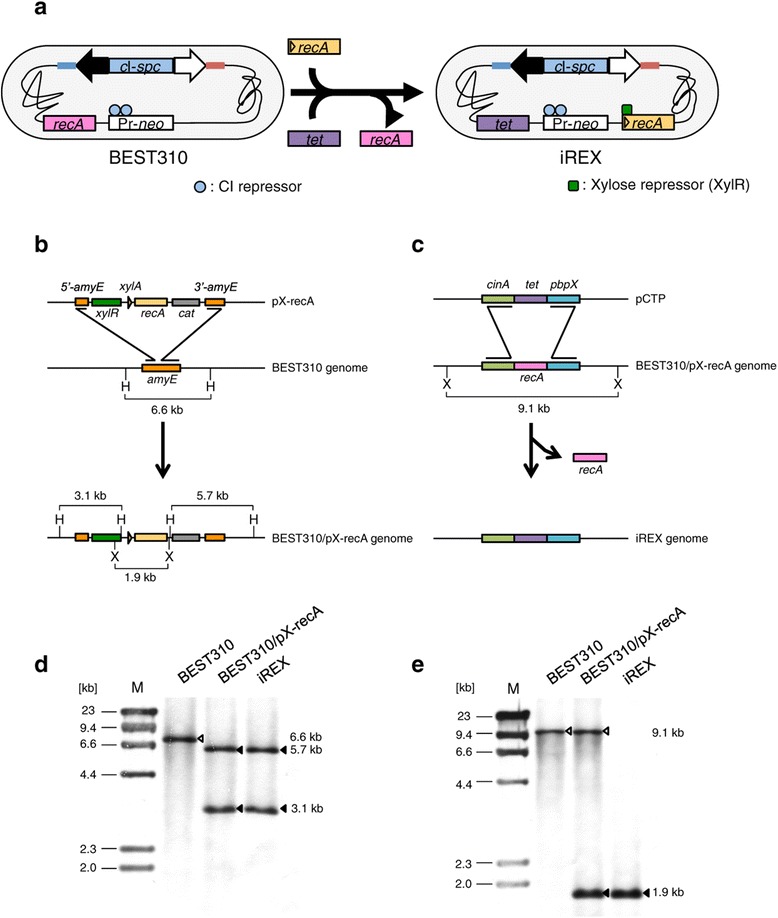
**Construction of the** ***recA*** **-inducible BGM vector system. (a)** The BEST310 and iREX constructs that possess two antibiotic resistance gene cassettes for BAC cloning: Pr-*neo*, a lambda Pr promoter fused to the neomycin resistance gene (*neo*), and *c*I-*spc*, which contains *c*I encoding the CI repressor protein, which binds to the Pr promoter, fused to the spectinomycin resistance gene (*spc*). The closed and open arrows indicate the BAC cloning site, and the red and blue lines indicate the pBR322 sequence. **(b)** The inducible *recA* expression cassette, pX-recA, was inserted at the *amyE* locus of the BEST310 genome via homologous recombination. *amyE* is not essential for the viability of *B. subtilis* [15]*. cat*, chloramphenicol acetyltransferase; H, HindIII; X, XhoI. **(c)** After introducing the pX-recA, the endogenous *recA* was replaced with the tetracycline resistance gene (*tet*) via homologous recombination. X, XhoI. **(d)** Southern blot analysis using an *amyE* probe indicated the correct insertion of pX-recA. The genomic DNA of the represented clones was digested with HindIII. The open arrowhead indicates the intact *amyE* in BEST310. The closed arrowheads indicate 5’-*amyE* and 3’-*amyE* divided by the insertion of pX-recA. **(e)** Southern blot analysis using a *recA* probe indicated the correct insertion of pCTP. The genomic DNA of the represented clones was digested with XhoI. The open arrowheads indicate the endogenous *recA*. The closed arrowheads indicate the inducible *recA* derived from pX-recA.

The inducible *recA* expression BGM vector (iREX) was constructed based on a BGM vector, BEST310, that was designed for BAC cloning [7], by introducing the inducible *recA* expression cassette followed by the targeted deletion of the endogenous *recA* (Figure 1a). For the inducible expression of *recA*, we used the gene expression cassette pX [15], in which the inducible promoter is regulated by xylose, and this cassette was designed to integrate into the *amyE* locus of *B. subtilis*. The inducible *recA* expression cassette pX-recA was constructed by cloning the *B. subtilis recA* into the BamHI site of pX and was integrated into the *amyE* locus of BEST310 to generate BEST310/pX-recA (Figure 1b). To delete the endogenous *recA* from BEST310/pX-recA, the targeted replacement of the endogenous *recA* with a tetracycline resistance gene (*tet*) was performed using pCTP, in which *tet* was inserted between the flanking sequences of the endogenous *recA* designated *cinA* and *pbpX* (Figure 1c). The resulting recombinant construct was designated as the inducible *recA* expression BGM vector, iREX. The insertion of pX-recA and the replacement of the endogenous *recA* with *tet* were confirmed by Southern blot analysis using an *amyE* probe and a *recA* probe (Figure 1d and e).

### Optimization of RecA inducing conditions in the iREX

**Figure 2:**
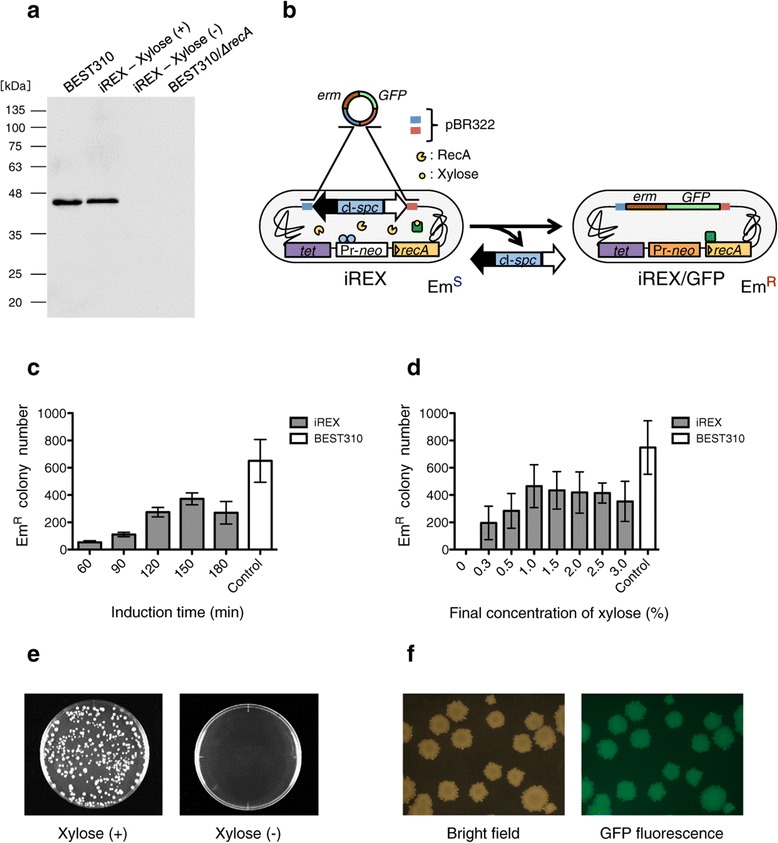
**Western blot analysis and optimization of RecA induction. (a)** Western blot analysis with an anti-RecA antibody indicated the expression of RecA in the presence of xylose. Remarkably, expression of RecA in the absence of xylose was strictly repressed. BEST310/*ΔrecA*, that was constructed by replacing the endogenous *recA* of BEST310 with *tet* of pCTP, was used as a negative control. **(b)** Schematic diagram of the cloning procedure. *erm*, erythromycin resistance gene; Em^S^, erythromycin sensitive; Em^R^, erythromycin resistant. **(c)** Numbers of erythromycin-resistant colonies under various induction times after addition of 1.0% xylose. Error bars, s.d. *n* = 3. **(d)** Numbers of erythromycin-resistant colonies under various xylose concentrations at 150 min induction. Error bars, s.d. *n* = 3. **(e)** Erythromycin-resistant recombinants were obtained only in the presence of xylose. The picture of xylose (+) is representative plate at a final xylose concentration of 1.0% at the induction time of 150 min. **(f)** All colonies formed on LB plates containing erythromycin were fluorescent due to their *GFP* gene.

To confirm the xylose-induced RecA expression, we first performed Western blot analysis. In the absence of xylose, there was no immunosignal for RecA detected from the iREX, indicating that the expression of *recA* was strictly repressed. In contrast, a signal for RecA was detected from the iREX in the presence of xylose as well as from the conventional BGM vector, BEST310. These results indicate that the expression of *recA* was strictly controlled by xylose (Figure 2a).

To optimize the conditions of RecA induction in the iREX, we examined the effects of xylose concentration and induction time on RecA activity. Because extracellular DNA can be integrated into the iREX genome by RecA activity, we evaluated RecA activity in terms of cloning efficiency. We measured cloning efficiency by transforming the iREX with the pSHINE2122, which contains the *GFP* gene and the erythromycin resistance gene (Figure 2b). pSHINE2122 is constructed from pBR322, allowing it to be integrated into the cloning site of the iREX, and recombinants can be screened based on GFP fluorescence and erythromycin resistance.

We first examined the induction time at a final xylose concentration of 1.0% (Figure 2c). The maximum number of erythromycin-resistant colonies was obtained at the induction time of 150 min. At this induction time, we next determined the optimal xylose concentration (Figure 2d). The number of erythromycin-resistant colonies plateaued at a final xylose concentration above 1.0%. Notably, there were no colonies in the absence of xylose (Figure 2d and e), indicating that the expression of *recA* was successfully repressed. The transformation of the iREX was also confirmed by GFP fluorescence. All the colonies that formed on the LB plates containing erythromycin were fluorescent (Figure 2f). The cloning efficiency of the iREX was approximately 60% of that of the original BGM vector, BEST310 (Figure 2d).

### Cloning of the BAC insert into the iREX

**Figure 3:**
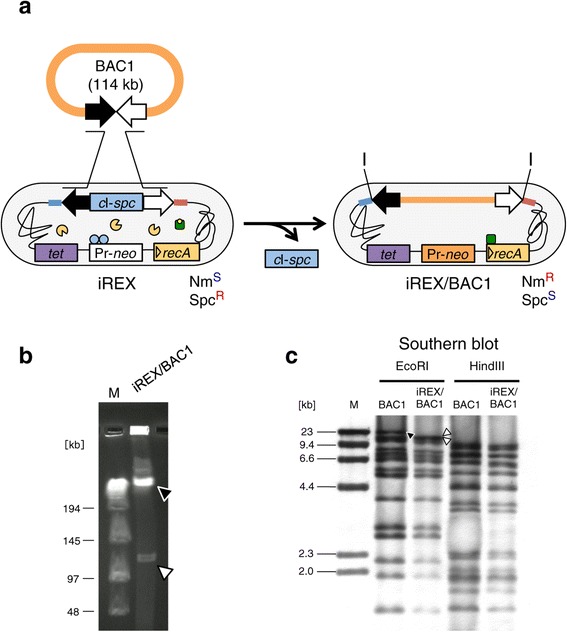
**Cloning of BAC1 into the iREX. (a)** One-step cloning of BAC1 into the iREX. Nm^S^, neomycin sensitive; Nm^R^, neomycin resistant; Spc^S^, spectinomycin sensitive; Spc^R^, spectinomycin resistant; I, I-PpoI recognition sequence. **(b)** iREX/BAC1 was digested with I-PpoI followed by CHEF gel electrophoresis. The BAC1 insert is indicated as an open arrowhead, and the BGM vector is indicated as a closed arrowhead. A lambda DNA concatemer was used as a size marker in lane M. **(c)** Original BAC1 and genomic DNA of the iREX recombinant were digested with EcoRI or HindIII and hybridized with the original BAC1 clone as a probe. Band patterns identical to the original BAC1 clones were confirmed in the iREX recombinant, except for the bands derived from the BAC end sequences. The closed arrowhead indicates a BAC1-specific signal, and the open arrowheads indicate BGM-specific signals. In lane M, lambda/HindIII fragments were used as a size marker.

One of the most attractive properties of the BGM vector system is its capacity to clone very large DNA fragments. To examine this important feature in the iREX, we conducted one-step cloning of BAC DNA into the iREX. The BAC clone, designated BAC1, carried a 114 kb mouse genomic DNA fragment containing two class I odorant receptor genes [10]. We transformed the iREX with BAC1 to construct iREX/BAC1 (Figure 3a). Briefly, the iREX is resistant to spectinomycin and sensitive to neomycin because the CI repressor represses the Pr-*neo* cassette. Once the BAC1 insert is cloned directly into the iREX genome via homologous recombination, the recombinants become resistant to neomycin and sensitive to spectinomycin due to the replacement of the *c*I-*spc* cassette with the BAC1 insert. Because two I-PpoI recognition sequences are introduced at the both ends of the cloning site, the cloned BAC1 insert could be excised by digesting the genomic DNA of the iREX recombinant with I-PpoI and analyzed by contour-clamped homogeneous electric field (CHEF) gel electrophoresis (Figure 3b). One of 20 candidate clones (resistant to neomycin and sensitive to spectinomycin) contained the BAC1 insert. The cloned insert was confirmed by Southern blot analysis using the original BAC1 clone as a probe (Figure 3c). The digestion patterns of iREX/BAC1 were identical to those of the original BAC clone, except for the fragments derived from the ends of the insert. Thus, the iREX was able to clone a giant DNA sequence of over 100 kb, similar to the conventional BGM vector system.

### Evaluation of the stability of the cloned DNA in the iREX

Previously, we demonstrated that the cloned BAC insert of the BGM vector could be inverted via homologous recombination between two homologous sequences in the reverse direction [10]. This inversion simply occurs during incubation due to the activity of endogenous RecA. Thus, the undesirable recombination of the cloned insert, such as by inversion, deletion, or rearrangement, can occur in the conventional BGM vector if the cloned insert has several homologous sequences. Because the expression of *recA* is tightly controlled in the iREX, it is possible that the iREX system can minimize such unnecessary recombination.

**Figure 4:**
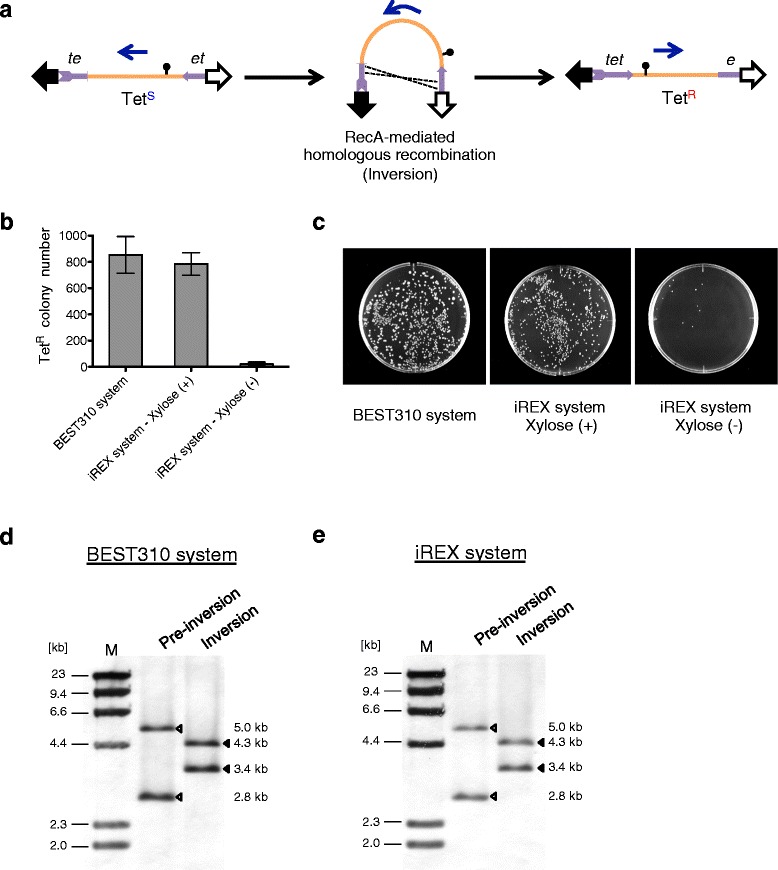
**Evaluation of the stability of the cloned DNA using inversion. (a)** Schematic diagram of the evaluation of the cloned DNA stability using inversion. Tet^S^, tetracycline sensitive; Tet^R^, tetracycline resistant. **(b)** Numbers of tetracycline-resistant colonies in the BEST310 system and the iREX system. Error bars, s.d. *n* = 3. **(c)** Many tetracycline-resistant recombinants were observed in the BEST310 system. In the iREX system, the same results were obtained in the presence of xylose because of the induced RecA. In contrast, few colonies were observed using the iREX in the absence of xylose. This result indicates that the cloned DNA insert of the iREX is stably maintained due to the strong repression of *recA*. (**d** and **e**) Southern blot analysis using a *tet* probe revealed changes in the sizes of the signals, indicating the inversion of the BAC1 insert. The genomic DNA of the represented clones was digested with BamHI. In lane M, lambda/HindIII fragments were used as a size marker.

To examine this, we first evaluated the stability of the cloned insert in terms of homologous recombination-mediated inversion using the *tet*-inversion cassette system [10]. Because the *tet*-inversion cassette system is dependent on the construction of the *tet*, we deleted *tet* in iREX/BAC1 by replacing *tet* with the *c*I-*bsr* cassette, which consisted of the CI repressor gene and the blasticidin S resistance gene. Then, two incomplete fragments of the tetracycline resistance gene, *te* (5’ end) and *et* (3’ end), which shared an overlapping region of approximately 1.1 kb, were inserted at the ends of the BAC1 sequences of iREX/BAC1 and of BEST310/BAC1. The recombination between the overlapping homologous sequences between the incomplete *tet* fragments resulted in the inversion of the cloned inserts as well as the acquisition of tetracycline resistance because of the formation of the complete *tet* (Figure 4a). Overnight cultures of iREX/BAC1 or BEST310/BAC1 with the *tet*-inversion cassette were spread on LB plates containing tetracycline, and the numbers of tetracycline-resistant colonies were counted. Notably, many tetracycline-resistant colonies were formed in the BEST310 system. The same tendency was observed in the iREX system in the presence of xylose. In contrast, there were few colonies in the iREX system in the absence of xylose, indicating that the cloned DNA insert was maintained much more stably in the iREX system than in the conventional BGM vector system (Figure 4b and c). The inversion of the inserts was confirmed by Southern blot analysis using a *tet* probe (Figure 4d and e).

A small number of colonies formed in the iREX system without xylose, which indicated that a residual recombinase activity remained in the iREX, most likely due to leaky expression of *recA* or other recombinase activity. To examine these possibilities, we constructed the *recA*-deficient strain, and performed the same experiment again. Because no tetracycline-resistant colonies were formed (data not shown), the small number of tetracycline-resistant colonies in the iREX system in the absence of xylose can be attributed to the leaky expression of *recA*.

### Application of the iREX to construct a transgene with multiple reporters

As shown in Figure 4, the existence of the homologous sequences in the cloned insert caused undesirable DNA rearrangement in the conventional BGM vector. To monitor the expression of multiple genes simultaneously, we often use the strategy of inserting multiple reporters using *IRES* bicistronic expression cassettes. It is possible that the multiple *IRES* sequences may mediate the homologous recombination and lead to the instability of the recombinants. To examine this possibility, i.e., unwanted recombination between two reporter sequences, we constructed transgenes with two bicistronic cassettes in both the iREX and the conventional BGM vector.

**Figure 5:**
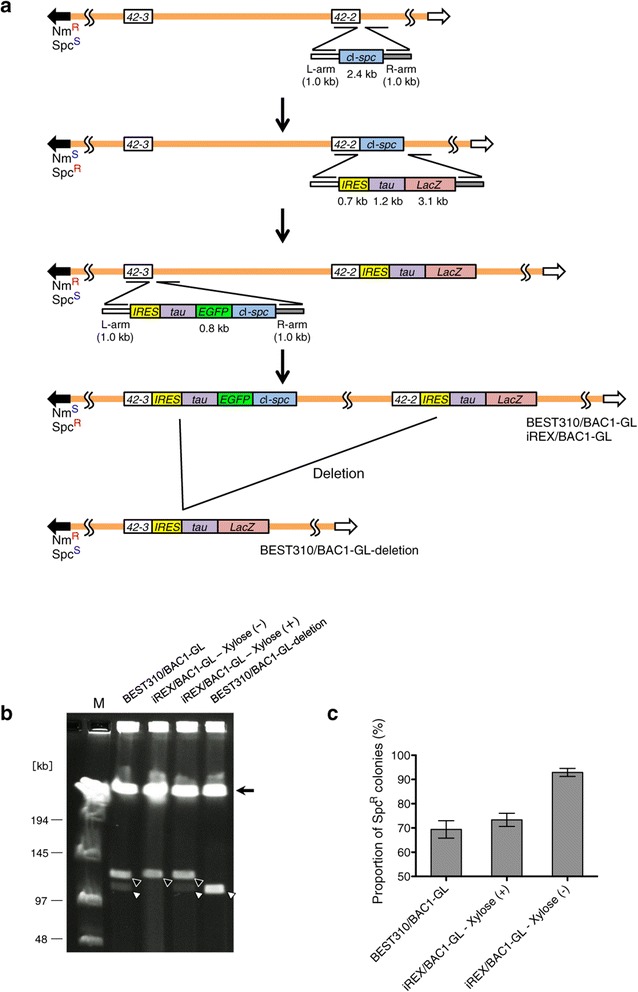
**Stable gene manipulation in the iREX by preventing the deletion. (a)** Schematic diagram of the insertion of *IRES*-*tauEGFP*-*c*I-*spc* and *IRES*-*tauLacZ* into *MOR42-3* and *MOR42-2*, respectively, and the assumed deletion at the *IRES*-*tau* sequences. **(b)** The signal derived from the deletion was observed in BEST310/BAC1-GL (open arrowhead). The same result was obtained in iREX/BAC1-GL in the presence of xylose because of the induced RecA. In contrast, the signal derived from the deletion was not observed for iREX/BAC1-GL in the absence of xylose due to the strong repression of *recA*. The closed arrowheads indicate the signals derived from the intact inserts of BEST310/BAC1-GL and iREX/BAC1-GL. BEST310/BAC1-GL-deletion, which was screened from BEST310/BAC1-GL culture by neomycin resistance and spectinomycin sensitivity, was used as a control of deletion. The BGM vector is indicated as a closed arrow. **(c)** The proportions of the spectinomycin-resistant clones containing two *IRES-tau* sequences were 69% and 73% in the BEST310/BAC1-GL and iREX/BAC1-GL with xylose, respectively. In contrast, the proportion of the spectinomycin-resistant clones was 93% in the iREX/BAC1-GL without xylose. Error bars, s.d. n = 3.

We inserted *IRES*-*tauEGFP*-*c*I-*spc* and *IRES*-*tauLacZ* 3 bp downstream of the stop codon of the class I odorant receptor genes of *MOR42-3* and *MOR42-2* in iREX/BAC1 and BEST310/BAC1 to generate iREX/BAC1-GL and BEST310/BAC1-GL, respectively (Figure 5a). Fusion of the microtubule-associated protein tau with the reporter protein enables the visualization of the axonal projections of neurons expressing the reporter gene. Both iREX/BAC1-GL and BEST310/BAC1-GL contain two homologous sequences of *IRES-tau*. First, we spread these recombinants on LB plates containing spectinomycin, and then we inoculated the formed colonies into liquid spectinomycin-free LB medium. The genomic DNA of the overnight cultures was digested with I-PpoI and analyzed using CHEF gel electrophoresis to examine the digestion pattern derived from the deletion. In BEST310/BAC1-GL, there was an additional signal, indicating that the deletion derived from the RecA activity had occurred (Figure 5b). The same result was obtained in the iREX/BAC1-GL with xylose due to the induced RecA. By contrast, no deletion signal was shown by the iREX/BAC1-GL in the absence of xylose. To examine the deletion event further, we quantified the event by estimating the proportions of the spectinomycin-resistant clones containing two homologous sequences of *IRES-tau.* The proportions of the intact clones were 69% and 73% in the BEST310/BAC1-GL and iREX/BAC1-GL with xylose, respectively (Figure 5c). On the other hand, the proportion of the intact clones was 93% in the iREX/BAC1-GL without xylose. These results indicate that the iREX can stably maintain the cloned DNA insert by preventing deletion at two homologous sequences.

Accordingly, the iREX is capable of handling large DNA fragments more stably than the conventional BGM vector system. In the YAC system, undesirable rearrangements also occur due to endogenous yeast recombination activity [4,11]. Thus, prevention of such undesirable rearrangements is necessary for the precise manipulation of large DNA fragments. In our previous study, the next generation DNA sequencing analysis of modified and reconstructed BGM clones confirmed that there were no mutations in cloned inserts with repetitive sequences, such as SINE and LINE [10]. However, in our current study, we observed deletions at the *IRES-tau* sequences during incubation only with the conventional BGM vector (Figure 5), suggesting that the existence of a certain homologous sequence can cause DNA rearrangement but that such rearrangements can be suppressed in the iREX.

## Conclusions

We developed a novel BGM vector, iREX, in which a xylose-inducible *recA* system was introduced. Western blot analysis and an evaluation of the stability of the cloned DNA insert demonstrate the considerable improvements in the stability of the cloned inserts that were achieved using the iREX due to the strict control of the expression of *recA*. The iREX can offer gene manipulation that is more stable than the conventional BGM vector and expand the utility of the BGM vector as a platform for engineering large DNA fragments.

## Methods

### Strains

The *B. subtilis* strain of the BGM vector, BEST310 [7], was used and modified to develop the inducible *recA* expression BGM vector (iREX) system. *B. subtilis* 168 *trpC2* [16] was also used for the PCR template to clone the *recA* and flanking sequences of the *recA* (*cinA* and *pbpX*).

### Construction of the inducible *recA* expression BGM vector (iREX)

To generate the iREX, we used the xylose-inducible gene expression cassette pX, which consists of the repressor gene *xylR* and the *xylA* promoter derived from the *Bacillus megaterium* operon for xylose utilization, followed by a multi-cloning site [15]. The *recA* was amplified by PCR (PrimeSTAR HS DNA polymerase, TaKaRa) using the genomic DNA of *B. subtilis* 168 *trpC2* as the template, and the PCR fragment was then cloned into the BamHI site of pX to construct pX-recA. The inducible *recA* expression cassette is flanked by 5’- and 3’- *amyE*; thus, the cassette can be inserted into the *amyE* locus of the BEST310 genome by homologous recombination. pX-recA was digested with ScaI (TaKaRa), and linearized pX-recA was introduced into the BEST310 genome via transformation. The recombinants were screened using chloramphenicol to obtain BEST310/pX-recA. To construct the endogenous *recA* deletion cassette, the flanking sequences of the endogenous *recA*, designated *cinA* (1.4 kb) and *pbpX* (1.4 kb), were amplified by PCR (PrimeSTAR HS DNA polymerase, TaKaRa) using the genomic DNA of *B. subtilis* 168 as the template. The PCR fragments were then cloned into the SalI-EcoRI site and the BamHI-SacII site of pBluescript II SK(+) to construct pCP. After these steps, an EcoRI-BamHI fragment of the tetracycline resistance gene from pBEST307 [17] was cloned into the EcoRI-BamHI site of pCP to generate pCTP. pCTP was digested with XhoI (TaKaRa), and the linearized pCTP was used for transformation to delete the endogenous *recA* of BEST310/pX-recA. The recombinants were screened using tetracycline to obtain the iREX. Primer sequences and PCR conditions are summarized in Additional file 1. The accuracy of the sequences generated by PCR was confirmed by DNA sequencing.

### Southern blot analysis of the BGM clones

Genomic DNA from the BGM clones was prepared using the liquid isolation method [18]. The genomic DNA was digested with HindIII, XhoI, EcoRI or BamHI (TaKaRa). The digested DNA was separated by CHEF gel electrophoresis followed by Southern blot analysis as described previously [10].

### Western blot analysis of RecA

The whole cell lysates of *B. subtilis,* BEST310 and the iREX were used for protein samples for the Western blot analysis. The competent cells of BEST310 were prepared as described elsewhere [18]. In the case of the iREX, xylose was added to the TF-II medium to a final concentration of 1% followed by 60 min incubation. The cells of BEST310 and the iREX were collected by centrifugation (1500 × g) at 4°C for 15 min and were washed twice by resuspending the pellet with 1 ml of PBS the first time and 0.7 ml the second time, followed by centrifugation (9500 × g) at 4°C for 2 min. After the final centrifugation, the cells were resuspended in PBS containing protease inhibitors (1 mM PMSF, 1.4 μM Pepstatin A, 0.3 μM Aprotinin, and 1 μM Leupeptin) in a final volume of 30 μL to give an OD_600_ of 50. The cells were lysed by repeated cycles of freezing and thawing (5 times). An equal volume of 2× SDS sample buffer [0.1 M Tris, 4.5% (wt/vol) SDS, 20% (vol/vol) Glycerol, 0.2% (vol/vol) Bromophenol blue] containing 12% (vol/vol) β-mercaptoethanol was added to the cell lysate, and aliquots were used for samples. Samples were heated for 5 min at 95°C before loading. Proteins were electrophoretically separated by SDS/PAGE on 12.5% (wt/vol) polyacrylamide gels and 5% (wt/vol) stacking gels and then transferred to nitrocellulose membranes (GE Healthcare). Each membrane was blocked in 5% (wt/vol) skimmed milk in TBS containing 0.05% (vol/vol) Tween 20, washed three times in TBST for 10 min each and then reacted with anti-RecA polyclonal antibody (Abnova, PAB15568) overnight at 4°C. Anti-RecA polyclonal antibodies were diluted 1:1,000 into 1% (wt/vol) skimmed milk in TBS-0.05% (vol/vol) Tween 20. Primary antibodies were detected using peroxidase-conjugated goat anti-rabbit IgG (GE Healthcare) with the ECL Western Blotting Detection System (GE Healthcare) and the Molecular Imager ChemiDoc XRS (Bio-Rad).

### Optimization of RecA induction in the iREX

The preparation of competent cells and the transformation of *B. subtilis*/BGM vectors were performed as described elsewhere [18]. To optimize the concentration of xylose, we added xylose to the growth medium to final concentrations of 0 to 3.0%. Briefly, xylose was added to the TF-II medium followed by 60 min incubation. When the xylose induction time was over 60 min, xylose was added to the TF-D medium again, followed by additional incubation. Transformation was performed at the end of the xylose induction by the addition of 500 ng of the pSHINE2122, which contains the *GFP* gene and the erythromycin resistance gene. pSHINE2122 harbors the erythromycin resistance gene instead of the chloramphenicol resistance gene found in pSHINE2121 [19,20]. The recombinants were screened for GFP fluorescence and erythromycin resistance. The colony number of the recombinants is an index of the induction efficiency.

### One-step transfer of the BAC insert into the iREX

The transfer of the BAC clone into the iREX was performed as described elsewhere except for the xylose-induction step [7,10]. The BAC clone, RP24-392H7 (designated BAC1), was purchased from the Children’s Hospital Oakland Research Institute. The BAC1 DNA was prepared by the alkaline lysis method and subsequent equilibrium centrifugation in a CsCl-ethidium bromide gradient [5]. The iREX was transformed with the purified BAC1 DNA in the presence of 1.0% xylose at 150 min induction.

### I-PpoI/CHEF analysis

The cloned BAC1 insert in iREX/BAC1 was analyzed by I-PpoI digestion followed by CHEF electrophoresis as described previously [10]. The inserts of BEST310/BAC1-GL and iREX/BAC1-GL were also analyzed by I-PpoI/CHEF analysis.

### Evaluation of the stability of the cloned DNA during incubation

To replace the *tet* in iREX/BAC1 with another antibiotic resistance gene, a *c*I-*bsr* cassette was inserted into the EcoRI site of pCP to generate pCCBP. pCCBP was linearized with XhoI (TaKaRa) and used for transformation to replace the *tet* of iREX/BAC1 with *c*I-*bsr*. After this modification, the 5’ side of the *tet* (*te*) and the 3’ side of the *tet* (*et*), which shared an overlapping region of approximately 1.1 kb, designated *e*, were sequentially inserted into both edges of the BAC1 insert of iREX/BAC1. The recombinants, which had both *te* and *et*, were screened using erythromycin and phleomycin. Similar constructs were prepared using the conventional BGM vector, BEST310, which includes the BAC1 insert (BEST310/BAC1). The recombinants of iREX/BAC1 were cultivated in LB broth at 37°C for 16 hours with or without xylose. The recombinant BEST310/BAC1 was also cultivated in LB broth at 37°C for 16 hours. A portion of the culture was spread onto an LB plate containing tetracycline. The inoculum was fixed according to the following formula: Inoculum (μL) = 100 / OD_600_.

### Construction of multiple-reporter transgenes

To construct iREX/BAC1-GL and BEST310/BAC1-GL, the *c*I-*spc* cassette was inserted 3 bp downstream of the stop codon of *MOR42-2* in iREX/BAC1 and BEST310/BAC1, followed by the replacement of the *c*I-*spc* cassette with the *IRES-tauLacZ* cassette and the insertion of the *IRES-tauEGFP-c*I-*spc* cassette 3 bp downstream of the stop codon of *MOR42-3*. To construct the *IRES*-*tauLacZ* cassette, the EcoRI site of the coding region of *LacZ* in the iTLZ-ACNF plasmid [21] was mutated by PCR mutagenesis, and an EcoRI-SpeI fragment of the *IRES-tauLacZ* cassette from the iTLZ-ACNF plasmid was inserted into the pT1 vector [10], whose NdeI site was deleted to construct pT1-iTLZ. The 1.0 kb left (L) and right 1.0 kb (R) arms for the targeted insertion of *c*I-*spc* into *MOR42-2* and the replacement of *c*I-*spc* with *IRES-tauLacZ* were prepared by PCR and contained sequences that were homologous to the upstream and downstream *MOR42-2* insertion sites, respectively. The L arm was first cloned into the SalI-EcoRI site of pT1-iTLZ, and the R arm was then cloned into the SpeI-SphI site to generate the *IRES*-*tauLacZ* cassette. The *c*I-*spc* cassette was constructed by inserting an EcoRI-SpeI fragment of *c*I-*spc* between the L arm and R arm. Primer sequences and PCR conditions are summarized in Additional file 1. The *IRES*-*tauEGFP*-*c*I-*spc* cassette was constructed previously [10].

### Estimation of the proportion of the spectinomycin resistant clones containing multiple reporter sequences

The iREX/BAC1-GL was cultivated in LB broth at 37°C for 16 hours with or without xylose. The BEST310/BAC1-GL was also cultivated as a control in LB broth at 37°C for 16 hours. A portion of the culture was spread onto an LB plate containing spectinomycin or neomycin. The spectinomycin-resistant colonies and neomycin-resistant colonies were counted and the proportion of the spectinomycin resistant colonies was calculated according to the following formula: (number of spectinomycin-resistant colony)/(number of spectinomycin-resistant colony + number of neomycin-resistant colony) × 100.

## Additional file

Additional file 1:
**Primer sequences and PCR conditions.** This file contains information of all primer sequences and PCR conditions used in this study for cloning and constructing homology arms, cassettes.

## Abbreviations

BGM: *Bacillus subtilis* genome
BAC: Bacterial artificial chromosome
YAC: Yeast artificial chromosome
iREX: Inducible *recA* expression BGM vector
CHEF: Contour-clamped homogeneous electric field
*IRES*: Internal ribosome entry site
EGFP: Enhanced green fluorescent protein
LacZ: Beta-galactosidase
